# EpiBasket: how e-commerce tools can improve epidemiological preparedness

**DOI:** 10.3402/ehtj.v6i0.19748

**Published:** 2013-10-31

**Authors:** Weijia Xing, Gilles Hejblum, Alain-Jacques Valleron

**Affiliations:** 1 Institut National de la Santé et de la Recherche Médicale, Paris, France; 2 Division of Infectious Disease, Key Laboratory of Surveillance and Early-warning on Infectious Disease, Chinese Center for Disease Control and Prevention, Beijing, China; 3 Université Pierre et Marie Curie-Paris 6, UMR-S 707, Paris, France

**Keywords:** data collection, disasters, disease outbreaks, emergency preparedness, epidemiology, information systems, questionnaires

## Abstract

**Background:**

Should an emerging infectious disease outbreak or an environmental disaster occur, the collection of epidemiological data must start as soon as possible after the event's onset. Questionnaires are usually built *de novo* for each event, resulting in substantially delayed epidemiological responses that are detrimental to the understanding and control of the event considered. Moreover, the public health and/or academic institution databases constructed with responses to different questionnaires are usually difficult to merge, impairing necessary collaborations. We aimed to show that e-commerce concepts and software tools can be readily adapted to enable rapid collection of data after an infectious disease outbreak or environmental disaster. Here, the ‘customers’ are the epidemiologists, who fill their shopping ‘baskets’ with standardised questions.

**Methods:**

For each epidemiological field, a catalogue of questions is constituted by identifying the relevant variables based on a review of the published literature on similar circumstances. Each question is tagged with information on its source papers. Epidemiologists can then tailor their own questionnaires by choosing appropriate questions from this catalogue. The software immediately provides them with ready-to-use forms and online questionnaires. All databases constituted by the different EpiBasket users are interoperable, because the corresponding questionnaires are derived from the same corpus of questions.

**Results:**

A proof-of-concept prototype was developed for Knowledge, Attitudes and Practice (KAP) surveys, which is one of the fields of the epidemiological investigation frequently explored during, or after, an outbreak or environmental disaster. The catalogue of questions was initiated from a review of the KAP studies conducted during or after the 2003 severe acute respiratory syndrome epidemic.

**Conclusion:**

Rapid collection of standardised data after an outbreak or environmental disaster can be facilitated by transposing the e-commerce paradigm to epidemiology, taking advantage of the powerful software tools already available.

When an outbreak of an emergent disease or an environmental disaster occurs, public health officials and researchers are expected to set up in real time the best possible epidemiological investigations to understand what happens, what the health consequences of the event are and how to mitigate them as quickly as possible (1).

The three conditions for this real-time epidemiological intelligence are real-time data collection, real-time methods of data analysis and real-time dissemination of results. The main obstacle of real-time epidemiology, addressed herein, is the first step in the process: data collection. Indeed, survey design and the elaboration of the corresponding questionnaires take time (2, 3): unsatisfactory questionnaires can be obtained quickly, but have to be modified a few days later; conversely, excellent questionnaires can be generated after lengthy meetings, but become available too late. Moreover, any important public health problem inherently involves a variety of actors in public health and research. All these players have their own agendas, hypotheses to test, stakeholders to comply with. Each of them designs a different survey, with different questionnaires (4). Finally, as a consequence, the information collected in the different surveys usually cannot be merged to provide a unified database, because the definitions and questionnaires used are not standardised (5, 6).

The best solution to overcome the above-outlined difficulties would be that a respected international or national agency, or a scientific society, provides THE questionnaire that everybody would agree to use in the case of an epidemiological disaster. That ‘top-down’ best solution approach is obviously unrealistic. One of the reasons is that epidemiological investigation is a research activity. Researchers like to design their own tools, and good quality research needs a diversity of approaches.

Herein, we describe software that achieves a ‘bottom-up’ approach aimed at accelerating and standardising data collection, while allowing epidemiologists to tailor their own questionnaires. The basic concept underlying the software was to revisit with an epidemiologist's eye the consumer's e-commerce model used daily. Indeed, not so long ago, the process of getting same-day delivery to your door of the best product, after an informed choice about all those available, was unimaginable. Today, when a customer wants to buy, for example, a new home-entertainment audiovisual system, the internet quasi-instantaneously provides all the necessary information on all possible TVs, DVD players, hard disks, speakers, and so on from a variety of manufacturers. He can choose his desired elements from each of these categories, put them in a shopping basket, and review and modify his choices before making his final decision. Then, he checks out and his system can be delivered in hours. The success of e-commerce led to the development of powerful software, some of it free, that we used in this project.

We show herein that this e-commerce model and its software can be readily adapted to epidemiology. In the proposed EpiBasket concept, epidemiologists are the ‘customers’. The ‘categories of products’ are the categories of questionnaire questions (e.g., the questions on the outcome of the household, life satisfaction, pre-validated scales for scoring stress). Information on which questions were used in the best high-profile papers published on similar events, information on all ‘products’ (i.e., questionnaire questions), will be available immediately. The epidemiologist devising his questionnaire will therefore be able to select from the ‘catalogue’ the best possible questions to meet his needs, put them in his ‘shopping basket’ and, once he has made up his mind, he ‘buys’ them and immediately gets his order, here the paper and/or the online version of the questionnaire. If the catalogue provides the best products on the ‘epidemiological market’, and if a community of researchers starts to collectively build and use this system, a positive byproduct should be the increased possibility of database interoperability because, in the future, epidemiologists working on the same subject will choose the questions for their questionnaires from the same catalogue.

In this article, we describe the general outline of the ‘EpiBasket’ concept in three sections: the construction of the catalogue of questions by the developer, the user's point of view and the software that was used. The proposed concept is illustrated with a prototype based on the Knowledge, Attitudes, Practices (KAP) studies conducted on the 2003 severe acute respiratory syndrome (SARS) epidemic (7). This prototype, whose detailed characteristics are described in the Supplementary file, can be assayed at http://www.epibasket.org

## Methods

The methodology used to stock the catalogue with questions and to tag each question with information of potential usefulness for the users is described in this section. The user's point of view and the software used are addressed in Results section.

### Construction of the catalogue of questions

The final goal is to provide users with a ‘catalogue’ of the ‘best’ questions needed to explore a given epidemiological issue (e.g., ‘the burnout of health workers during the outbreak’ or ‘the changes of attitudes towards immunisation in the general population during an outbreak’). A basic assumption underlying the EpiBasket project is that most of the essential questions that are needed today were posed in the past, and that those providing the results published in the best scientific journals should certainly be considered as the best possible candidate questions. Three steps were defined to stock the catalogue with questions.

The first was to identify the ‘variables of interest’ that must be acquired to investigate a given issue. This identification relied on a systematic review of the pertinent papers published in the domain. To facilitate the extraction of those variables, we supposed that they were mentioned in the tables and figures of the papers analysed in the systematic review. The manual extraction of those variables that we performed to build the prototype could subsequently be automated, with natural language-analysis tools (8) ‘reading’ the tables, figures and their corresponding legends. A relational database comprising all extracted variables was constituted. It allows tracking of the context of the variable, for example, to immediately find the sets of variables that are usually used together.

In a perfect world, the questionnaires used in each reviewed study would be made available to readers. In the real world, very few authors provide their questionnaires, hence the ‘question’ had to be inferred from the ‘variable’. Therefore, the second stage was to deduce the ‘question’ from the ‘variable’ identified in the previous step. For example, the questionnaire may have asked the date of birth, while the paper may report the corresponding results in a table showing age-class frequencies. In addition, EpiBasket can propose a recommendation for a preferred coding, with the aim of maximising the chances of interoperability among databases constituted with EpiBasket-derived questionnaires and their responses.

Third, the formulation of questions was generalised, when possible, to make them reusable in broader situations than those in the source papers. It was accomplished by replacing words or patterns found in the reviewed papers by ‘root terms’ that the user would replace with the specific term that fits the event of interest. For example, the question ‘In the case of a SARS outbreak, would you avoid going to the cinema?’ provides a template for asking questions regarding avoidance behaviours during an outbreak. Now, using ‘root terms’, it is formatted as ‘In the case of #$DISEASE outbreak, would you avoid going to #$PLACE?’, where #$DISEASE and #$PLACE are the root terms to be instantiated by the user selecting this question template, for example, ‘In the case of a cholera outbreak, would you avoid going to a restaurant?’ So far, six root terms have been implemented in the current EpiBasket prototype: ‘#$DISEASE’, ‘#$DRUG’, ‘#$NUMBER’, ‘#$PERIOD’, ‘#$PLACE’ and ‘#$SOMEONE’. For example, the specific questions ‘Did you wear a mask in a public place?’, ‘Did you wear a mask at work?’ and ‘Did you wear a mask during a flight?’ were reframed as a single generic formulation, ‘Did you wear a mask #$PLACE?’.

In addition to the questions extracted from the literature, a ‘User suggestions’ category was added to store user-proposed questions.

### Tagging of the questions

Each question in the catalogue has tags aimed at helping the user construct his questionnaire.

#### General tags

Categories to which the question belongs, time of its use (before, during and/or after the epidemiological event considered), type(s) of population(s) studied (general population, patients, risk groups, healthcare workers), MeSH description of the term(s) used in the question.

#### References in literature

List of the published papers analysed that used the question (full article references, links to PubMed and Google Scholar, journal impact factors [IF]).

#### ‘Value’ of a question

Each question is also tagged with a ‘value’, the equivalent of a price in e-commerce. The underlying assumption is that, when the user will have to choose among several questions (e.g., choosing among several scales available for scoring burnout), the one with the best ‘value’ will be retained. Since such a logical view is likely to be followed by most EpiBasket users, EpiBasket should facilitate better standardisation of questionnaires. At present, we arbitrarily decided to give to a question the value defined as the ‘Best IF’, that is, the highest journal IF encountered among all papers using it. This choice assumes that, if the user has to choose between two burnout scales, the one previously published in a high-profile journal will be preferred. As a consequence of this valuing system, all demographic variables (e.g., age, sex) get a high value (because they are used in all papers, including those published in journals with very high IF). IF can be systematically obtained from the Journal Citation Reports database, for which a fee must be paid. However, most journals provide their IF on their websites and we used those values in the prototype (see the Supplementary file). The value of 0.000 was arbitrarily assigned to questions published in journals not indexed in the Journal Citation Reports database. Researchers not wanting to take these IF into account can just ignore the ‘values’ indicated when they build their questionnaire.

#### Keywords

Each question was associated with at least one keyword. These keywords are given on the EpiBasket homepage as a tag cloud (with term size proportional to term frequency). Whenever the user clicks on a given cloud term, all questions associated with it are listed.

#### Time of study/population studied

Each paper from which the questions were extracted concerns a study conducted at a given time, either during the outbreak or after it, and a given population type. This information is available to the user to help him to identify the relevant questions for his own study.

#### Related questions

Each EpiBasket question provides the list of the other questions jointly asked in the papers that used it. EpiBasket also provides the list of questions in the catalogue that share at least one of the keywords associated with question.

#### Information on the questionnaire developers

To maximise the likelihood of compatibility among questionnaires, we paralleled the e-commerce approach, according to which shoppers know when they buy, for example, a book, what other books other customers also bought. Here, we implemented the function ‘users who selected this question also selected … <*names of the other questions*>’.

## Results

**Fig. 1:**
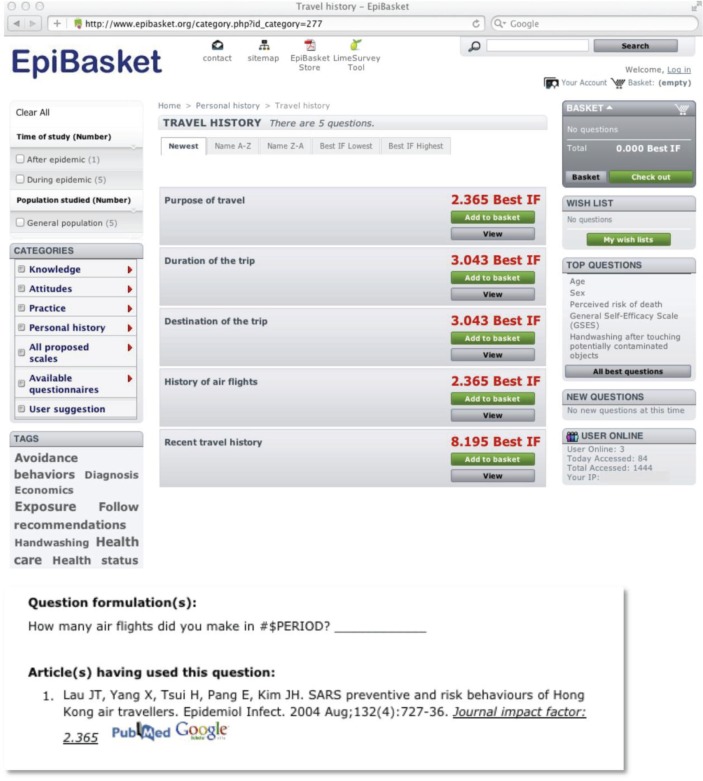
Example of an EpiBasket window (see http://www.epibasket.org for a demonstration). In this example, the user chose the subcategory ‘Travel history’ in the ‘Personal history’ category (left column). Five questions are proposed in this subcategory (middle of the window). If the user is also interested in the related question dealing with ‘past flight history’, clicking on question 4 will open a window giving the reference to the paper with the highest impact factor (IF) in the database that posed that question, and the exact question formulation used in that paper.

Composing a questionnaire is viewed, from the user's point of view, as ‘shopping for’ questions, that is, filling a ‘basket’ with the questions he wants to eventually have in the questionnaire. However, while the order in which the items composing the shopping basket were added does not matter in a usual consumer's purchase, here the questions composing a questionnaire must be appropriately ordered. The EpiBasket software helps the user draft a questionnaire and to exchange his draft (that we called a ‘wish list’) with his collaborators until he decides to obtain a ready-to-use paper questionnaire, for a paper-and-pencil survey and/or an electronic file for an online survey. In addition, EpiBasket includes several tools enabling user–user and user–Editorial Committee exchanges to promote collaborative epidemiology. Fig. 1 gives a snapshot of one of the windows. A demonstration of the different EpiBasket features of can be tried at http://www.epibasket.org.^1^ These features are described below in more detail.

### Building the draft of the questionnaire

First, the user must select the candidate questions pertaining to the event of interest: questions according to categories of the questions, time of study, study population and so on. For example, the user may want to consult all the questions belonging to the ‘Knowledge’ category, concerning healthcare workers and to be collected during the outbreak, which would require three clicks. Because questions are labelled with keywords and linked to papers in which they were initially mentioned, suggested associations of potential appropriate questions can be easily accessed.

### Building a wish list and filling a shopping basket

The user prepares a ‘wish list’ that will eventually be transformed into a final ‘buy’. A given user may constitute different wish lists, which may be shared with colleagues. These wish lists allow epidemiologists to discuss questionnaire contents upstream before impression of the final product. Finally, the user selects questions in the wish list to fill his ‘shopping basket’, which becomes the final questionnaire. The order in which selected questions are added to the basket will be the order of the questions in the final document.

### Obtaining the questionnaire

When the user validates his basket, EpiBasket automatically generates the corresponding questionnaire in three formats: XML, pdf and csv. XML is the most generic output of EpiBasket, as it is the most popular encoding standard for machine-readable document files. Appropriate parsing of an EpiBasket XML output file greatly facilitates further importation of the questions (including the proposed answers) constituting the generated questionnaire in any popular relational database-management system. The pdf format is available for epidemiologists wanting to conduct a paper-and-pencil survey. The csv format is proposed to those planning an online survey: users can directly import their csv file and initiate the corresponding survey online in EpiBasket (http://www.epibasket.org/limesurvey/admin/index.html). The user can also install on his own server LimeSurvey (9), freeware designed for building and deploying online surveys, and run the survey on this server. The electronic questionnaire imported in LimeSurvey may be modified by the user, if desired.

**Fig. 2:**
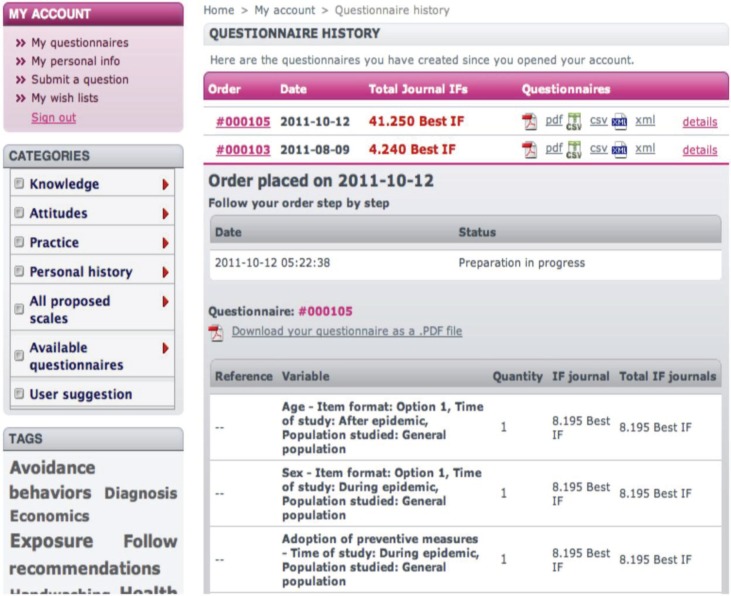
The questionnaire manager in EpiBasket. A user can store and edit his successive questionnaires or drafts of questionnaires listed at the top of the middle panel. Each of the questionnaires is available in pdf (for a paper questionnaire), in csv (for an online survey using the link to LimeSurvey), or in XML (the most generic EpiBasket output). The preview of the questions is available immediately.

The generated questionnaires are stored in the user's ‘My questionnaires’ section (Fig. 2). The user has permanent access to any of the questionnaires ordered since his initial registration in the EpiBasket site.

### Promoting interactions among epidemiologists

Several tools were developed in EpiBasket to encourage collaborations among epidemiologists and facilitate users’ comments and suggestions on the methods. More specifically, users can comment on each catalogue question, just as e-commerce customers express their opinions on the products being sold. Users can also propose new questions by filling out an electronic form, recommend word changes in a question and suggest coding of the responses. They can also save questionnaires that they find relevant for outbreak or environmental disaster epidemiology in the ‘Epibasket store’. This possibility was illustrated in the available EpiBasket prototype with a selection of questionnaires on coronavirus outbreaks.

### Software

For the present proof-of-concept demonstration of EpiBasket, we used free open-source code. The main components of the EpiBasket-prototype development are described below.

The EpiBasket application was built in the Eclipse 3.6 software-development environment (10), using PHP5 language. Development was based on the importation and adaptation/customisation of the freeware part of PrestaShop (11), an open-source PHP5-based developed application devoted to the building of online e-commerce sites. PrestaShop, a full-featured cross-platform shopping basket package, was written in PHP5 with an underlying MySQL relational database-management system. PrestaShop can be deployed in the server-side supporting PHP5 and can be customised through the inclusion of modules proposed at the PrestaShop site (free or not, many supplemental module blocks were developed and are available for enhancing PrestaShop functions), or through a developer's initial code modification (i.e., code suppression/correction/addition).

The main adaptation/customisation features for developing EpiBasket were the following. An initial group of modifications was made to cope with the epidemiological specificity of EpiBasket: the catalogue's offered ‘e-commerce products’ are ‘epidemiological questions’. All ‘orders’ and ‘invoices’ are replaced by ‘questionnaires’. Moreover, user account-information features used in e-commerce, such as (‘my credit slips’, ‘my vouchers’, ‘my personal info’, and so on) required substantial modifications/suppressions/additions. For example, in EpiBasket, the ‘orders’ attached to a given user are ‘my questionnaires’, that is, those ordered since his initial registration in the EpiBasket site. We also developed a specific source code to allow a user account to propose the implementation of a new question in the EpiBasket catalogue. All these adaptations required numerous and substantial code-source changes of the original e-commerce software.

A second group of modifications concerns the relational associations between tags and questions, and between related questions to facilitate the user's navigation interface to explore questions. To do so, the following PrestaShop modules were implemented: Reposition module to allow a drop-down presentation of question categories and subcategories; Filter search community edition to filter questions according to our desired criteria; Product bought by other people to enable users to examine which questions were associated in questionnaires in previous questionnaires built by EpiBasket users; and cy-related products which provides a list of those questions sharing keyword similarities with the question currently displayed.

A third group of specific developments concerned the EpiBasket questionnaire outputs. The open-source quexml tool (12) was implemented in the Eclipse EpiBasket project and was used to generate a questionnaire according to the encoding XML 1.0 standard scheme. The XML questionnaire template was in turn used to generate pdf or csv versions. A questionnaire written in XML can easily be converted to paper (pdf format), enabling rapid deployment of paper-and-pencil surveys, whereas the converted csv file can be directly imported into the LimeSurvey freeware, enabling rapid deployment of an online survey. LimeSurvey (13) is an open-source online-survey application written in PHP, based on a MySQL, PostgreSQL or MSSQL database, and distributed under the GNU General Public License. It is particularly user friendly, as it enables users to develop and publish surveys, and collect responses, without doing any coding (14). LimeSurvey also offers the possibility of designing telephone surveys with the computing-assisting telephone interviewing (CATI) standard through the use of the quexs tool that was not considered in the current EpiBasket prototype.

## Discussion

Herein, we described a prototype that, by adapting WEB tools and e-commerce concepts to epidemiology, should accelerate the creation of an epidemiological questionnaire at the time of a public health emergency. The strength of our approach is that it relies on and takes full advantage of past knowledge and experiences in similar events. The goal is not only avoiding that epidemiologists ‘reinvent the wheel’ when they are faced with situations similar, or partly similar, to those the past. It also strives to indirectly encourage more standardised and better quality questionnaires. Another indirect attribute of the proposed approach is to promote interactions among epidemiologists. Indeed, despite informatics advances, which make it easy, at least in theory, to devise databases easily sharable and accessible from any place, the fact is that, in practice, this is not yet reality (15). The main reason is likely to be found in human factors. However, the tool we present has integrated the potential to stimulate collaborative approaches. For example, the ‘EpiBasket store’ can host a public depository for existing questionnaires, which are very rarely made available by authors in their papers.

In this prototype, we chose to exemplify the EpiBasket concept with KAP studies after an outbreak because they are quite similar to those studies done after an environmental disaster. They represent a small part of the epidemiological investigations done during/after an outbreak and a full-size EpiBasket should provide questions for all fields concerned. After the 2003 SARS epidemic, we identified (7) at total 10 fields other than KAP belonging to three categories: Investigation and Surveillance (four fields: description of the outbreak, search for causative agents, transmission studies, risk factors), case management (five fields: clinical presentation, diagnosis, treatment and medical interventions, prognosis, medical decision-making), and prevention and control. Thus, the EpiBasket prototype described herein is far from what a full-size ‘EpiBasket’ could be. The design and computer implementation of that ideal project would require a workforce (i.e., epidemiologists and software engineers) that exceeds the resources of a single research group. We think the software aspect is likely the easiest, as it was shown here that the efforts already made in the profitable field of e-commence can be relatively easily adapted to epidemiology. The future editorial and organisational aspects are much more difficult to foresee. The editorial work covers the definitions of the means applied to ‘value’ the questions. For the prototype, we chose the best journal IF among published papers on similar events that used the question. It is also possible, for example, that an Editorial Committee would prefer a mechanism according high values to questions submitted by experts in the field. In that scenario, coding recommendations should certainly be made by subcommittees. One of the organisational issues concerns copyrighted questionnaires, particularly those that are available only for a fee. The current EpiBasket version does not address such issues.

Finally, because a major objective of EpiBasket is to support collaborative epidemiology, additional tools could be added to the users’ community at very little informatics cost: for example, a registry of epidemiological investigations on outbreaks or public health disasters, where epidemiologists could register their new studies before publication with a brief description, authors’ names, affiliations and addresses. The difference with the current database on clinical trials (16) would be that epidemiologists would be free to register or not.

The key message of the EpiBasket prototype is that the informatics tools that are used daily and successfully by lay people, like e-commerce and WEB 2.0 applications, have great potential to enhance the quality and timeliness of epidemiological studies, especially in the context of investigation of outbreaks or other public health events requiring a real-time, quality approach. We also think that our proposed approach can help the desired trend towards more transparency, more data sharing and more collaborations among epidemiologists.

## Footnotes

1 A password can be obtained upon request from the corresponding author (A-J V).

## References

[1] Fineberg HV, Wilson ME (2009). Epidemic science in real time. Science.

[2] Boynton PM, Greenhalgh T (2004). Selecting, designing, and developing your questionnaire. BMJ.

[3] Burns KE, Duffett M, Kho ME, Meade MO, Adhikari NK, Sinuff T (2008). A guide for the design and conduct of self-administered surveys of clinicians. CMAJ.

[4] Schilling LM, Kozak K, Lundahl K, Dellavalle RP (2006). Inaccessible novel questionnaires in published medical research: hidden methods, hidden costs. Am J Epidemiol.

[5] Kleppner D, Sharp PA (2009). Research data in the digital age. Science.

[6] Olsen J (1998). Epidemiology deserves better questionnaires. IEA European Questionnaire Group. International Epidemiological Association. Int J Epidemiol.

[7] Xing W, Hejblum G, Leung GM, Valleron A-J (2010). Anatomy of the epidemiological literature on the 2003 SARS outbreaks in Hong Kong and Toronto: a time-stratified revie. PLoS Med.

[8] Cunningham H, Maynard D, Bontcheva K, Tablan V (2002). GATE: an architecture for development of robust HLT applications. Proceedings of the 40th Annual Meeting of the Association for Computational Linguistics, July 2002.

[9] LimeSurvey download page http://www.limesurvey.org/en/download.

[10] Eclipse http://www.eclipse.org/downloads/packages/eclipse-classic-362/heliossr2.

[11] PrestaShop http://www.prestashop.com/.

[12] queXML http://quexml.sourceforge.net/.

[13] LimeSurvey http://limesurvey.org/.

[14] Turner BL, Haygarth PM (2001). Biogeochemistry. Phosphorus solubilization in rewetted soils. Nature.

[15] Editorial (2006). Dreams of flu data. Nature.

[16] Clinical Trials http://clinicaltrials.gov/.

